# A Great Hospital's Work under Trying Conditions

**Published:** 1920-06-05

**Authors:** 


### THE LONDON HOSPITAL.

### A Great Hospital's Work Under Trying Conditions.

The chief business of the quarterly Court of Governors, held on June 2, was to consider the serious financial position of the London Hospital. This matter is dealt with on another page, but some interesting details of the weekly cost of maintaining a great hospital were laid before the Governors. The following figures show the amount of weekly expenditure on certain items: Meat costs approximately £216 per week; fish, £98; butter and bacon, £124; eggs, £84; milk, £320; bread, £44; grocery, £130; vegetables, £58; drugs, £160; bandages, £84; instruments, £80; coal, £226; electricity, £52; salaries and wages, £1,800. It was stated that the position in which the hospital finds itself is such that, unless the public helps in the emergency, the whole hospital, or part of it, will have to be closed at a time when the hospital is not only full, but when there is a list of 791 persons waiting their turn for admission.

### Wages and Hours of Employees.

A conference has been held at the Ministry of Labour with regard to the wages and hours of workers. The matter has been before the Court of Arbitration, whose decision is awaited. The Committee are strongly of opinion that a charity should not be run on the charity of its employees, who ought to be paid reasonable wages and to work for reasonable hours, and they have agreed to stand by the decision of the Court.

### Resignation of a Physician and Surgeon.

The resignations of Dr. Percy Kidd and Mr. Jonathan Hutchinson were referred to. On the unanimous recommendation of the Committee, these officers were appointed consulting physician and surgeon respectively. The services of Dr. Kidd and Mr. Hutchinson were alluded to in warm terms, as well as their unfailing kindness and courtesy to every patient and to every employee of the hospital.

On the recommendation of the Committee, Dr. R. A. Rowlands and Dr. John Parkinson, both of whom have been medical registrars, were appointed assistant physicians of the hospital, and Mr. E. C. Lindsay, who has been a surgical registrar, was appointed an assistant surgeon. The assistant physicians take the place of Dr. F. J. Smith and Dr. Percy Kidd, and Mr. Lindsay fills the vacancy made by the retirement of Mr. Jonathan Hutchinson.

The medical and surgical units have started work. Dr. Ellis and Dr. Riddoch have been appointed assistants in the medical unit, and Messrs. Lindsay, Perrin, and Neligan assistants in the surgical unit.

### Increased Grant from the Local Government Board.

The grant from the Local Government Board for the treatment of venereal disease has been increased from £4,500 per annum to £6,000 per annum.

Miss Hosking, who for fourteen years has been in charge of the preliminary training-school at Tredegar House, is retiring through ill-health. She is to be pensioned at £110 per annum.

### Death of Sir Henry Burdett, K.C.B.

The Committee made sympathetic reference to the death of Sir Henry Burdett, K.C.B., who had for so many years been a staunch supporter of the voluntary system and a true friend of all voluntary hospitals. He was always ready to be of assistance to the hospital, and constantly made sympathetic and appreciative references to it in his paper The Hospital.

